#  Anti-inflammatory Effects of Oxymatrine Through Inhibition of Nuclear Factor–kappa B and Mitogen-activated Protein Kinase Activation in Lipopolysaccharide-induced BV2 Microglia Cells 

**Published:** 2013

**Authors:** Xiao-Qiao Dong, Quan Du, Wen-Hua Yu, Zu-Yong Zhang, Qiang Zhu, Zhi-Hao Che, Feng Chen, Hao Wang, Jun Chen

**Affiliations:** *Department of Neurosurgery, The First Hangzhou Municipal People’s Hospital, Nanjing Medical University. *

**Keywords:** Microglia, Oxymatrine, Nuclear factor kappa-B, Mitogen-activated protein kinase, Inflammation

## Abstract

Oxymatrine, a potent monosomic alkaloid extracted from Chinese herb *Sophora japonica *(Sophora flavescens Ait.). possesses anti-inflammatory activittyes. This study was designed to investigate the effects of oxymatrine on nuclear factor–kappa B (NF-*κ*B) and mitogen-activated protein kinase (MAPK)-dependent inflammatory responses in lipopolysaccharide (LPS)-activated microglia. In this paper, BV2 microglia were pretreated with different concentrations of oxymatrine (1, 10 and 20 μg/mL) for 30 min as followed by stimulation with LPS (1 μg/mL) for different times (30 min, 1 h, 3 h, and 6 h). Concentrations of nitric oxide (NO), prostaglandin E_2_ (PGE_2_), tumor necrosis factor-alpha (TNF-*α*), interleukin-1beta (IL-1*β*) and interleukin-6 (IL-6) in supernatant, mRNA levels of inducible nitric oxide synthase (iNOS) and cyclooxygenase-2 (COX-2), cytosolic inhibitor of kappa B-alpha (I-κB*α*) and phospho- I-κB*α *and nuclear p65 protein levels, and the phosphorylations of MAPK molecules such as extracellular-signal-regulated kinase (ERK) 1/2, p38 MAPK and c-Jun N-terminal kinase (JNK) were determined. It was shown that oxymatrine inhibited the productions of NO, PGE2, TNF-*α*, IL-1*β *and IL-6, attenuated the mRNA levels of iNOS and COX-2, suppressed the phosphorylation of I-κB*α *in cytosol, decreased the nuclear levels of p65, and also blocked ERK, p38 and JNK pathway in LPS-stimulated BV2 microglial cells in a dose-dependent manner. According to the results; It is suggested that oxymatrine may attenuate inflammatory responses of microglia and could be potentially useful in modulation of inflammatory status in the brain disorders.

## Introduction

Oxymatrine (C_15_H_24_N_2_O); (OMT), a potent monosomic alkaloid extracted from Chinese herb *Sophora japonica *(Sophora flavescens Ait.), has a tetracyclic quinolizine structure, this alkaloid possesses activities of anti-inflammaty, immune regulatioory, antivirus, anticancer, antiapoptosis and antifibrosis activity, and is originally used for the treatment of acute or chronic viral hepatitis (1-7). In the recent years, OMT studies have focused gradually on its therapeutic effect against other inflammatory diseases. OMT is proven to protect ischemic and reperfusion injury in lung, intestine and heart via anti-inflammatory process (8-12). Furthermore, Liu and collaborators investigated the potential neuroprotective role of oxymatrine in cerebral ischemia and found that OMT reduces infarct volume through the decreasing of nuclear factor kappa-B (NF-κB) expression (13, 14). In addition, it was evidenced that OMT protected the brain from damage caused by middle cerebral artery occlusion through down-regulation of mitogen-activated protein kinases (MAPKs) (15). We also found that OMT could suppress the synthesis of tumor necrosis factor –alpha (TNF-*α*), interleukin-1beta (IL-1*β*) and interleukin-6 (IL-6) after traumatic brain injury via NF-κB pathway (16). However, there has been a lack of studies regarding the effects of OMT on inflammation in an *in-vitro *model of brain inflammation.

Microglia are specialized macrophages and widely distributed in the brain (17, 18). Microglia comprise approximately 10–20% of the total glial cells in the adult central nervous system (19). Microglia may play a dual role. Participating in host defense of the brain as well as acting as phagocytes to engulf tissue debris and dead cells. Microglia can also augment neuroinflammation by secreting various neurotoxic and inflammatory mediators in chronic brain diseases. causing neuronal death and demyelination (20-23). In response to brain injury or neuroinflammatory stimuli, microglia may overproduce inflammatory and/or cytotoxic factors, including nitric oxide (NO), prostaglandin E_2_ (PGE_2_), IL-1*β*, IL-6 and TNF-a. These factors are characteristic of various neurodegenerative diseases, including Alzheimer’s disease, Parkinson’s disease, trauma, multiple sclerosis and cerebral ischemia. Reduction of inflammatory mediators in microglia could attenuate the severity of these disorders (24, 25). These results indicate that activated microglia are a major cellular source of inflammatory and/or cytotoxic factors that cause neuronal damage in the central nervous system. Therefore, controlling microglial activation has been considered to be an important therapeutic strategy for the treatment of many neuroinflammatory diseases (26).

Lipopolysaccharide (LPS), a bacterial endotoxin, initiates a number of major cellular effects that play critical roles in the pathogenesis of inflammatory responses and has been employed to induce microglial activation during infection by Gram-negative bacteria. LPS stimulation of the microglia is therefore a useful model for the study of mechanisms underlying neuronal injury by various inflammatory and neurotoxic factors released by activated microglia (27, 28). LPS activates NF-kB and MAPKs family, which are classified into at least three components: extracellular signal-regulated kinases (ERKs), c-Jun N-terminal kinase (JNK), and p38 MAPK (29), which have been implicated in the release of immune-related cytotoxic factors such as inducible nitric oxide synthase (iNOS), cyclooxygenase-2 (COX-2), and inflammatory cytokines (30, 31).

In the present study, we attempted to elucidate the antiinflammatory potential of OMT by investigating the effect of OMT on the inflammatory response induced by LPS in murine microglial BV-2 cells. To further investigate the underlying mechanisms, the involvement of NF-kB and MAPKs was also examined. The present study provides information revealing OMT as a potential candidate with antiinflammatory actions and suggests a scientific basis for further investigation of OMT against neuroinflammatory conditions.

## Experimental

*Compounds*

OMT was purchased from Shanxi Huike Botanical Development Company Limited (Shanxi, China). OMT was dissolved in dimethyl sulfoxide (Sigma, MO, USA) to yield a 10 mg/mL stock solution and was diluted to the indicated concentrations in the experiments.

*Cell culture*

BV2, a murine microglial cell line, which is a suitable model for *in-vitro *study of microglia, was used in this study. The cells were grown in a flask (75 cm^2^) and washed with phosphate buffered saline (PBS) solution twice and then treated with trypsin-EDTA (TE; Biowest, Nuaille, France) in PBS for 3 min at 37**°**. The TE was inactivated by equal volume of 1×fetal bovine serum (FBS; HyClone, Utah, USA). The culture was centrifuged at 1000 rpm at 4**° **for 5 min and the pellet was resuspended in 10 mL of Dulbecco’s Modified Eagle’s Medium (DMEM, Sigma, MO, USA) containing 10% FBS and 1% antibiotic antimycotic cocktail (Sigma, MO, USA). The cells were counted using a hematocytometer and approximately 2×10^6^ cells were plated into each flask containing 10 mL of 10% FBS in DMEM and grown at 37**° **and 5% CO_2_ in an incubator. The cells were subcultured every 2-3 days. For experiments, the BV2 cells were maintained in DMEM without antibiotics or FBS for the required periods of treatment (Basic medium). 

*Treatment of cell culture*

2×10^6^ cells were plated onto cell culture dishes and grown in 10% DMEN/FBS with antibiotics overnight. On the following day, the cells were washed twice with PBS, transferred to basic medium and pretreated with different concentrations of OMT for 30 min as followed by stimulation with LPS (1 μg/mL; Sigma, MO, USA) for different times (30 min, 1 h, 3 h, and 6 h) in the incubator. The control was taken as cells grown in basic medium for the same time periods. The cells and supernatant collected were used for analysis. Each experiment was performed in three independent experiments.

*Nitrite assays (Griess assay)*

NO levels in the culture supernatants were measured by a Griess reaction. After cells (5×10^5^ cells/mL) were stimulated in 24 wells for 24 h, 100 μL of each cultured medium was mixed with the same volume of the Griess reagent (1% sulfanilamide/0.1% *N*-(1-naphthyl)-ethylenediamine dihydrochloride/2.5% H_3_PO_4_). NO concentration was determined by measuring the absorbance at 540 nm with a Vmax 96-well microplate spectrophotometer. Nitrite concentration was calculated with reference to a standard curve of sodium nitrite generated by known concentrations. Results were expressed as μM.

*Western blot analysis*

Cells were suspended in lysis buffer (1% Triton X-100, 1% deoxycholate, and 0.1% NaN_3_) and incubated for 30 min on ice. For nuclear extraction, cells were lysed with NE-PER^TM^ (Pierce, Rockford, IL, USA). Protein concentrations were determined (Bio-Rad, Hemel, Hempstread,UK) and same quantity of proteins were resolved on 10% gels, and then transferred to nitrocellulose membranes (Millipore, Billerica, MA, USA). The membrane blots were blocked with 5% milk in TBS-T (o.1 M Tris-HCl, pH 7.4, 0.9% NaCl, 0.1% Tween-20) and incubated with primary antibodies against *β*-actin (Santa Cruz Biotech, Santa Cruz, CA), inhibitor of kappa B-alpha (I-κB*α*), phospho-I-κB*α *(p-I-κB*α*), p65 NF-*κ*B, the phosphor (p) - or total forms of ERK 1/2, p38 MAPK and JNK (Cell Signaling Technology Inc., Beverly, MA, USA). The membranes were subsequently incubated with peroxidase-conjugated affinity goat anti-rabbit IgG (Sigma, MO, USA) and detected by chemiluminescence detection system (ECL, Amersham, Bershire, UK). Relative intensity was presented.

*Enzyme-linked immunosorbent assay (ELISA)*

The levels of TNF-*α*, IL-1*β*, IL-6 and PGE2 in the culture media were determined by ELISA. ELISA kits from R&D Systems (Minneapolis, MN) were employed for the measurement of TNF-*α*, IL-1*β *and IL-6, and a kit from Cayman Chemical (Ann Arbor, MI) was employed for the measurement of PGE_2_ in accordance with the manufactures’ instructions. Results were expressed as pg/mL.

*Reverse transcription-polymerase chain reaction analysis (RT-PCR)*

Total RNA was prepared from BV-2 cells by using the Trizol® reagent (Invitrogen Corporation, Carlsbad, CA, USA) according to the manufacturer’s protocol. RNA (2 μg) of each sample was used for synthesizing cDNA through inverse transcription; 1 μL of cDNA was used to carry out PCR amplification. Primers were synthesized by Shanghai Sangon Biological Engineering Technology Company Limited. Correctness of the gene order was proved in GenBank. Primers included 5’- CTGCAGCACTTGGATCAGGAACCTG-3’ (forward) and 5’-GGGAGTAGCCTGT GTGCACCTGGAA-3’ (reverse) for iNOS, 5’-TTGAAGACCAGGAGTACAGC-3’ (forward) and 5’-GGTACAGTTCCATGACATCG- 3’ (reverse) for COX-2, as well as 5’-AGCCATGTACGTAGCCATCC-3’ (forward) and 5’-GCTGTGGTGGTGA AGCTGTA -3’ (reverse) for *β*-actin. PCR amplification of the resulting cDNA template was conducted by using the following conditions for 45 (*β*-actin), 36 (COX-2) or 27 (iNOS) cycles. After an initial denaturation step at 95°C for 15 min, temperature cycling was initiated. Each cycle consisted of denaturation at 94°C for 15 sec, annealing at 60°C for 25 sec, and elongation at 72°C for 20 sec (*β*-actin). After an initial denaturation step at 95°C for 5 min, temperature cycling was initiated. Each cycle consisted of denaturation at 94°C for 30 sec, annealing at 57 °C for 45 sec, and elongation at 72°C for 30 sec (COX-2). After an initial denaturation step at 95°C for 5 min, temperature cycling was initiated. Each cycle consisted of denaturation at 94°C for 45 sec, annealing at 60°C for 45 sec, and elongation at 70°C for 1 min (iNOS). RT-PCR products (5 μL) were analyzed by 2% agarose gel electrophoresis. The gray scale of the electrophoresis strip was scanned by an ultraviolet photometry (UVP) gel imaging system. The relative expression of products was normalized to *β*-actin mRNA; data were analyzed with an image analysis system.

*Cytotoxicity assay*

The cell viability of the cultured cells was determined by measuring the reduction of 3-(4, 5-dimethylthiazol-2-yl)-2, 5-diphenyltetrazolium bromide (MTT) to formazan. Briefly, cell were seeded and treated with different concentrations of OMT (1, 10, 20, 50 and 100 μg/mL) and 0.5 mg/mL amount of MTT solution was added to each well. After incubation for 2 h at 37°C and 5% CO_2_, the supernatants were removed and the formed formazan crystals in the viable cells were dissolved in dimethyl sulfoxide. The absorbance at 570 nm was determined using a microplate reader (Molecular device, USA). Data were expressed as percent change of controls. Each experiment was performed in three independent experiments.

*Statistical analysis*

All data in this study were presented as mean ± standard error. Statistical analysis was performed with SPSS 10.0 software (SPSS Inc., Chicago, USA). All data were analyzed by one-way analysis of variance followed by Least-significant Difference test. Significance levels were set at *P*<0.05.

## Results and Discussion

*Effect of OMT on BV2 microglial cells viability*

**Figure 1:**
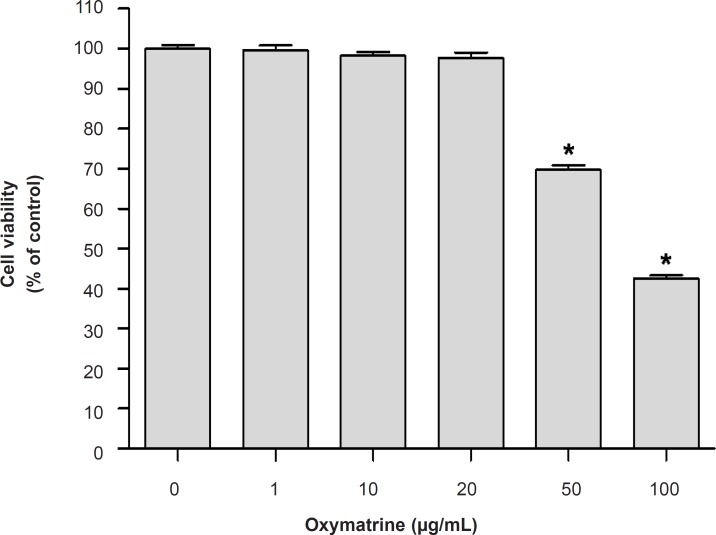
Effect of OMT on BV2 microglial cells viability. Data represent mean ± standard error of three independent experiments. *p < 0.05 vs. cells alone

To determine whether treatment of OMT only caused toxicity on BV2 microglial cells, the MTT assay was carried (Figure 1). OMT at concentrations 1, 10 and 20 μg/mL had no effect on the viability of BV2 microglial cells and used for further studies. 

*Effect of OMT on LPS-induced production of NO and expression of iNOS mRNA in BV2 microglial cells*

**Figure 2:**
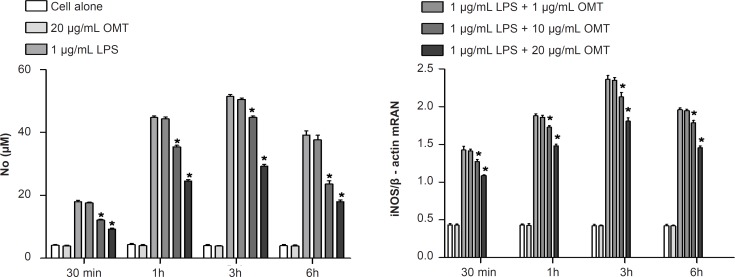
Effect of OMT on LPS-induced production of NO and expression of iNOS mRNA in BV2 microglial cells. Data represent mean ± standard error of three independent experiments. *p < 0.05 vs. LPS alone

To investigate the effect of OMT on production of inflammatory mediators, NO, in LPS-stimulated microglia, we measured NO concentrations by Griess assay. OMT decreased LPS-induced NO production in BV2 microglial cells with a dose-dependent manner (Figure 2). To elucidate the mechanism responsible for the inhibitory effect of OMT on NO production, we next investigated the levels of iNOS mRNA by RT-PCR. OMT inhibited the levels of iNOS mRNA (Figure 2) induced by LPS stimulation in BV2 microglial cells with a dose-dependent manner.

*Effect of OMT on LPS-induced production of PGE* *2* *and expression of COX-2 mRNA in BV2 microglial cells*

**Figure 3:**
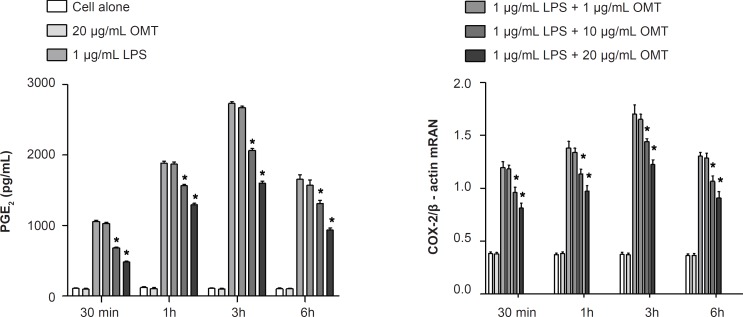
Effect of OMT on LPS-induced production of PGE_2 _and expression of COX-2 mRNA in BV2 microglial cells. Data represent mean ± standard error of three independent experiments. *p < 0.05 vs. LPS alone

To investigate the effect of OMT on production of inflammatory mediators, PGE2, in LPS-stimulated microglia, we measured PGE2 concentrations by ELISA. OMT decreased LPS-induced PGE_2_ production in BV2 microglial cells with a dose-dependent manner (Figure 3). To elucidate the mechanism responsible for the inhibitory effect of OMT on PGE_2_ production, we next investigated the levels of COX-2 mRNA by RT-PCR. OMT inhibited the levels of COX-2 mRNA (Figure 3) induced by LPS stimulation in BV2 microglial cells with a dose-dependent manner. 

*Effect of OMT on LPS-induced production of inflammatory cytokines in BV2 microglial cells*

**Figure 4:**
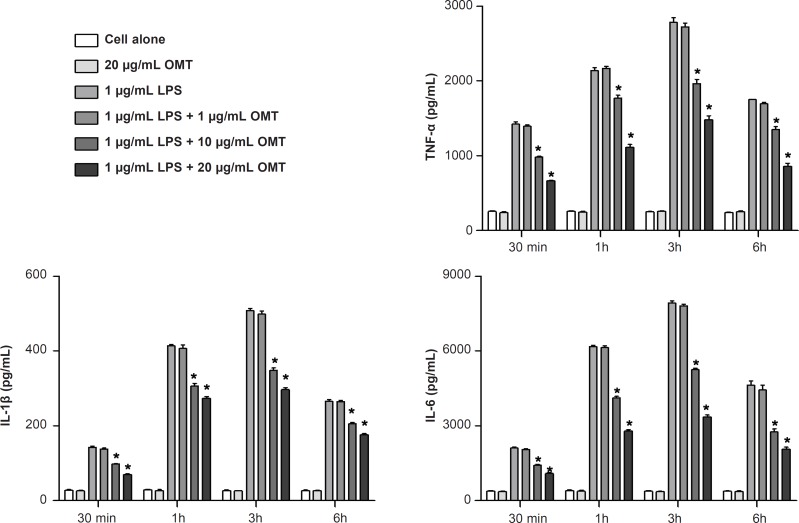
Effect of OMT on LPS-induced production of inflammatory cytokines in BV2 microglial cells. Data represent mean ± standard error of three independent experiments. *p < 0.05 vs. LPS alone.

To investigate the effect of OMT on the production of inflammatory cytokines, we measured the concentrations of TNF-*α*, IL-1*β *and IL-6 by ELISA. OMT attenuated LPS-induced productions of TNF-*α*, IL-1*β *and IL-6 in BV2 microglial cells in a dose-dependent manner (Figure 4). 

*Effect of OMT on LPS-stimulated NF-κB activation in BV2 microglial cells*

**Figure 5:**
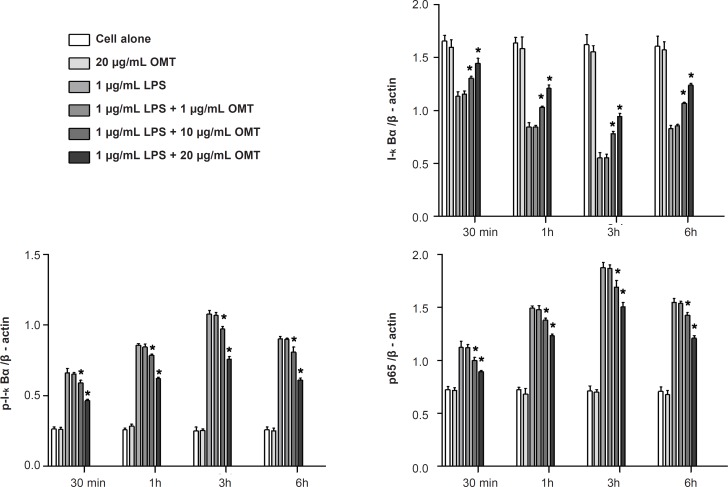
Effect of OMT on LPS-stimulated NF-κB activation in BV2 microglial cells. Data represent mean ± standard error of three independent experiments. *p < 0.05 vs. LPS alone

To determine whether OMT blocked the NF-κB pathway which is implicated in the transcriptional regulation of inflammatory mediators in activated microglia, we analyzed the cytosolic levels of I-κB*α *and p-I-κB*α *as well as nuclear levels of NF-κB p65 subunit by Western blot. OMT suppressed the phosphorylation of I-κB*α *in cytosol and the nuclear levels of NF-κB p65 in LPS-stimulated BV2 microglial cells in a dose-dependent manner (Figure 5). 

*Effect of OMT on LPS-stimulated MAPK activation in BV2 microglial cells*

**Figure 6:**
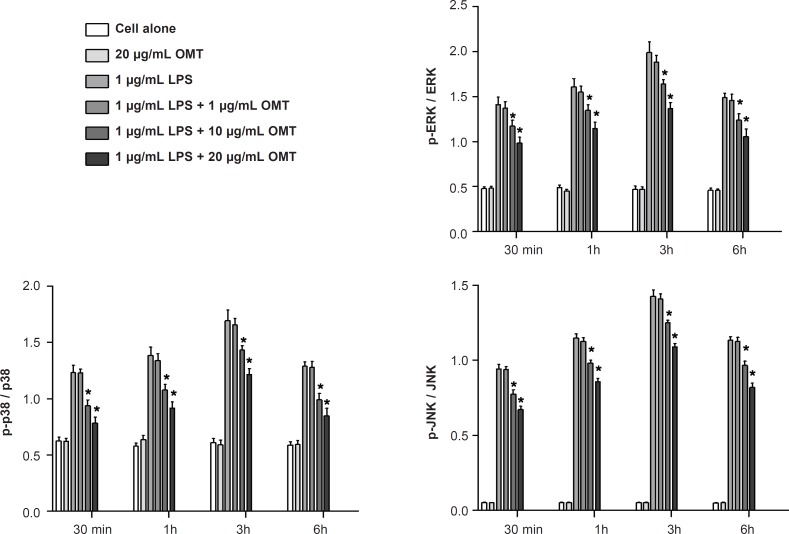
Effect of OMT on LPS-stimulated MAPK activation in BV2 microglial cells. Data represent mean ± standard error of three independent experiments. *p < 0.05 vs. LPS alone

To determine whether the inhibitory effect of OMT on gene expression of inflammatory mediators occurred through alteration of MAPK activity, we investigated the phosphorylations of MAPK molecules such as ERK 1/2, p38 MAPK and JNK by Western blot. OMT attenuated LPS-induced the phosphorylations of ERK 1/2, p38 MAPK and JNK in BV2 microglial cells in a dose-dependent manner (Figure 6). 

Microglia are potential sources and targets of relevant neuroprotective factors as well as neurotoxins because their uncontrolled activation may contribute to neurotoxicity in brain disorders (20-23). The activated microglia-derived inflammatory mediators such as NO, PGE_2_, TNF-*α*, IL-1*β *and IL-6 has long been involved with neuroinflammation in neurological disorders (24, 25). Thus, intervention of microglial activation will become a therapeutic target for the treatment of many inflammatory diseases (26).

NO is known to be involved in the processes of central nervous system inflammation. iNOS is the key enzyme for NO production, and quantitatively induced in activated glial cells after exposure to various stimulators (32). Therefore, agents with ability to inhibit iNOS expression are potentially beneficial in the treatment of conditions associated with NO overproduction including inflammatory diseases (33). In this study, OMT inhibited LPS-induced NO production through suppression of iNOS expression in BV2 microglial cells in a concentration-dependent manner. This data indicates that OMT acts by regulating NO generation, and that it could be a suppressor of microglial activation.

After LPS stimulation, microglia showed an increase in the inducible COX-2, which seems to be the crucial enzyme for the production of PGE2 during inflammation, and contributes to the development of chronic neurological disorders such as stroke, multiple sclerosis, Alzheimer’s disease and Parkinson’s disease, (34, 35). In this study, OMT inhibited LPS-induced PGE_2_ production by reducing the COX-2 expression in BV2 microglial cells in a concentration-dependent manner. This result indicates that the inhibition of PGE_2_ production by OMT might be due to the suppression of COX-2 up-regulation in activated microglia.

Inflammatory cytokines such as TNF-*α*, IL-1*β *and IL-6 are three main inflammatory cytokines that are produced by activated microglia during central nervous system inflammation. In the central nervous system, a number of stimuli, such as LPS, *β*-amyloid and traumatic brain injury have been shown to abundantly produce TNF-*α*, IL-1*β *and IL-6 (36, 37). Overproduction of inflammatory cytokines from activated microglial cells has a detrimental effect on neuronal cells (20, 21). This study investigated whether OMT inhibits LPS-induced production of inflammatory cytokines in BV-2 cells. Our data suggest that OMT inhibited LPS-induced production of TNF-*α*, IL-1*β *and IL-6 in activated BV-2 cells in a dose-dependent manner. This result indicates that OMT is able to modulate the activities of inflammatory cytokines in activated microglia.

NF-kB is known as a pleiotropic regulator of various genes involved in the production of many inflammatory cytokines and enzymes related to the inflammatory process. NF-kB is a central regulator of microglial responses to activating stimuli, including LPS. NF-kB, as a consequence of its key role in several pathologic conditions, is a major drug target in a variety of diseases. Early regulation occurs in the cytoplasm with the activation of I-κB*α *and subsequent I-κB*α *phosphorylation. This phosphorylation results in I-κB*α *degradation, thus allowing NF-kB translocation to the nucleus (38-40). NF-kB activation is critical for the expression of various cytokines, iNOS and COX-2 in microglia in response to LPS (41, 42). We showed that OMT prevents LPS-induced phosphorylation of I-κB*α*, p65 nuclear translocation and NF-kB binding to a consensus sequence in a concentration-dependent manner. Therefore, inhibition of NF-kB signaling pathways in microglia by OMT might result in the down-regulation of inflammatory mediators, thereby resulting in an antiinflammatory effect.

Various intracellular signalling pathways are involved in inflammatory mediator expression. MAPKs are a group of signalling molecules that also appear to play key roles in inflammatory processes. Previous studies have shown that activation of MAPKs have a significant effect on the regulation of COX-2, iNOS, and inflammatory cytokine gene expression by controlling the activation of NF-kB in microglia (43-45). So, it is possible that neuroprotective mechanisms are associated with inhibition of MAPKs in activated BV2 microglia. Therefore, we investigated the effect of OMT on LPS-stimulated phosphorylation of ERK 1/2, JNK and p38 MAPK in BV2 microglia. The present study demonstrated that OMT inhibited LPS-induced phosphorylation of ERK 1/2, p38 MAPK and JNK in activated microglia in a concentration-dependent manner. This finding indicates that the down-regulation of inflammatory mediators by OMT in activated microglia is at least partly due to blocking of the MAPK pathway. 

Our results show that OMT treatment of BV2 microglia results in decreased levels of inflammatory mediators following LPS stimulation, and the inhibitory effect of OMT was mediated by inhibition of NF-κB, ERK 1/2, p38 MAPK and JNK activation in activated microglia. Given the fact that OMT shows an antiinflammatory property in vivo, this findings suggest that OMT may provide a beneficial effect in the treatment of inflammatory brain damages.
